# Supplementary material for: The Antimicrobial Peptide D-CONGA-Q7 Eradicates Drug-Resistant E. coli by Disrupting Bacterial Cell Membranes
Source: Biology (Basel). 2025 Feb 21;14(3):226. doi: 10.3390/biology14030226 (PMC11940214; doi:10.3390/biology14030226)
Supplement: Supplementary file 1 [file biology-14-00226-s001.zip › supplementary materials.pdf]

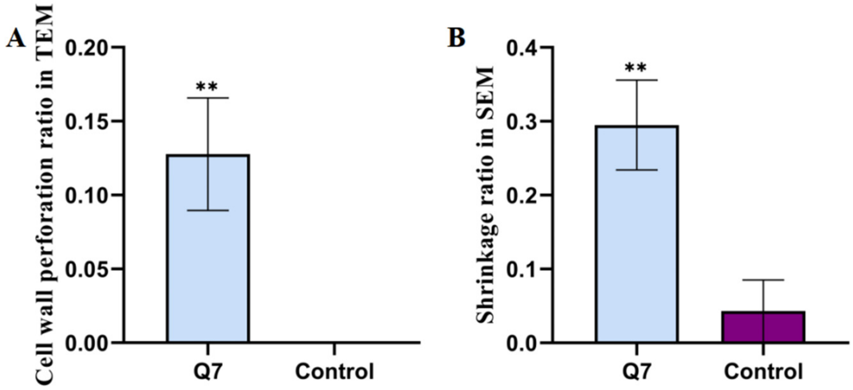

Figure S1. Q7 exerts bactericidal effects by altering the permeability of the inner and outer membranes and compromising the integrity of the bacterial cell wall. (A,B) Proportion of cell membrane wrinkled under scanning electron microscopy and proportion of membrane ruptured under transmission electron microscopy in bacteria treated with Q7. Data in panels A,B represent three biological replicates (n = 3), expressed as mean ± SD. Statistical significance compared to control is indicated as follows: ** $p < 0.01$.
